# Regional Gray Matter Density Associated with Cognitive Reflectivity–Impulsivity: Evidence from Voxel-Based Morphometry

**DOI:** 10.1371/journal.pone.0122666

**Published:** 2015-03-24

**Authors:** Ryoichi Yokoyama, Takayuki Nozawa, Hikaru Takeuchi, Yasuyuki Taki, Atsushi Sekiguchi, Rui Nouchi, Yuka Kotozaki, Seishu Nakagawa, Carlos Makoto Miyauchi, Kunio Iizuka, Takamitsu Shinada, Yuki Yamamoto, Sugiko Hanawa, Tsuyoshi Araki, Hiroshi Hashizume, Keiko Kunitoki, Mayu Hanihara, Yuko Sassa, Ryuta Kawashima

**Affiliations:** 1 Department of Functional Brain Imaging, Institute of Development, Aging and Cancer, Tohoku University, Sendai, Japan; 2 Japan Society for the Promotion of Science, Tokyo, Japan; 3 Smart Ageing International Research Center, Institute of Development, Aging and Cancer, Tohoku University, Sendai, Japan; 4 Division of Developmental Cognitive Neuroscience, Institute of Development, Aging and Cancer, Tohoku University, Sendai, Japan; 5 Division of Medical Neuroimaging Analysis, Department of Community Medical Supports, Tohoku Medical Megabank Organization, Tohoku University, Sendai, Japan; 6 Department of Nuclear Medicine and Radiology, Institute of Development, Aging and Cancer, Tohoku University, Sendai, Japan; 7 Human and Social Response Research Division, International Research Institute of Disaster Science, Tohoku University, Sendai, Japan; 8 Graduate Schools for Law and Politics, The University of Tokyo, Bunkyo, Tokyo, Japan; 9 Faculty of Medicine, Tohoku University, Sendai, Japan; The University of New South Wales, AUSTRALIA

## Abstract

When faced with a problem or choice, humans can use two different strategies: “cognitive reflectivity,” which involves slow responses and fewer mistakes, or “cognitive impulsivity,” which comprises of quick responses and more mistakes. Different individuals use these two strategies differently. To our knowledge, no study has directly investigated the brain regions involved in reflectivity–impulsivity; therefore, this study focused on associations between these cognitive strategies and the gray matter structure of several brain regions. In order to accomplish this, we enrolled 776 healthy, right-handed individuals (432 men and 344 women; 20.7 ± 1.8 years) and used voxel-based morphometry with administration of a cognitive reflectivity–impulsivity questionnaire. We found that high cognitive reflectivity was associated with greater regional gray matter density in the ventral medial prefrontal cortex. Our finding suggests that this area plays an important role in defining an individual’s trait associated with reflectivity and impulsivity.

## Introduction

Human problem-solving canonically requires the adoption of one of two cognitive strategies. Throughout the literature, these strategies [1, 2] have been widely categorized as reflective and intuitive [3], explicit and implicit [4], controlled and automatic [5], or system 1 and system 2 [3]. Furthermore, it has been suggested that the utilization of these strategies varies among individuals [4].

In psychology, the two problem-solving approaches have been identified as types of cognitive style and are classically referred to as reflectivity and impulsivity [6–8]. The cognitive reflectivity strategy is seen in individuals who are slow responders and commit fewer mistakes, whereas cognitive impulsivity is observed in individuals who respond quickly, committing more mistakes [9]. Importantly, impulsivity as a reference to cognitive style in psychology should not be compared to impulsivity as it is used in psychiatric studies such as addiction research [10]. This is because impulsivity has a negative connotation in the field of psychiatry since it has been defined as a trait related to poor notion, premature execution, undue risk, or inappropriate actions that often result in undesirable consequences [11]. In contrast, impulsivity as a concept in psychology does not have a negative connotation [12, 13]; rather, it is considered necessary to maintain a balance between the rapidness and accuracy of an action. This view is supported by several findings indicating that psychiatric and psychological measurements involve different aspects of impulsivity [13–16].

Recently, magnetic resonance imaging (MRI) has been used as a tool to investigate how white and grey matter (GM) structure can predict individual differences in a variety of human cognitive functions [17]. For example, previous psychiatric studies have been able to identify a relationship between the orbitofrontal cortex (OFC) volume and impulsivity [18, 19]. However, to our knowledge, no study has directly investigated the brain structures involved in cognitive reflectivity*–*impulsivity. As previously mentioned, impulsivity in psychiatry is a different concept than impulsivity in psychology; therefore, we assume that their neural basis will also be different.

Previous neuroimaging studies have identified several regions of the brain responsible for reflectivity and impulsivity. For example, the reflective system, which includes the dorsolateral prefrontal cortex (DLPFC), anterior cingulate, insula cortex, and hippocampus, is thought to play a central role in reflectivity [20], while the impulsive system, which includes the striatum and amygdala, is associated with impulsive behavior [20]. The neural system that integrates information from both the reflective and impulsive systems has been identified [21, 22]; we termed it “the integration system” and have used this term hereafter. For example, the ventromedial prefrontal cortex (vmPFC) is thought to be part of the integration system because it is associated with aspects of both reflectivity and impulsivity; some studies have categorized the vmPFC as a reflective system [20], while others have categorized it as an impulsive system [5].

However, the brain structure responsible for representing individual differences in cognitive reflectivity–impulsivity is still unknown. For the neural basis of cognitive reflectivity–impulsivity, we focus on two primary, non-mutually-exclusive possibilities: (1) individual differences in cognitive reflectivity–impulsivity could be mediated by brain regions involved in reflective and/or impulsive processing or (2) individual differences in cognitive reflectivity–impulsivity can be represented in the integration system. On the basis of previous studies, if the first possibility is true, then the reflective system and/or the impulsive system may be responsible for the individual differences in cognitive reflectivity–impulsivity. On the other hand, if the second possibility is true, then the integration system may be responsible for the individual differences in cognitive reflectivity–impulsivity. Thus, our first hypothesis is that the reflective system and/or the impulsive system is responsible for individual differences in cognitive reflectivity–impulsivity, while our second hypothesis is that the integration system mediates these differences.

To test our hypotheses, we investigated the association between individual differences in cognitive reflectivity–impulsivity and regional GM density (rGMD) by using voxel-based morphometry (VBM) [23]. For assessing cognitive reflectivity–impulsivity, we used a cognitive reflectivity–impulsivity questionnaire [24]. Further, in order to adjust for the effects of intelligence on brain structure, the Raven’s Advanced Progressive Matrix (RAPM) test [25] was conducted and used for an analysis.

## Methods

### Ethics Statement

In accordance with the Declaration of Helsinki (1991), written informed consent was obtained from the participants prior to their participation in the present study. The Tohoku University School of Medicine Ethics Committee approved the study protocol.

### Subjects

Seven hundred and seventy-six healthy, right-handed individuals (432 men and 344 women; 20.7 ± 1.8 years) participated in this study as part of an ongoing project investigating associations among brain region, cognitive function, age, genetics, and daily habits [26–34]. Data generated from the subjects in this study will likely be used in other studies unrelated to the theme of the current investigation, and some of the subjects who participated in this study became subjects of intervention studies (only psychological and imaging data recorded before the intervention was used in this study). All subjects were university, college, or postgraduate students or subjects who had graduated one year before the study onset. All participants had normal vision and no history of neurological or psychiatric illness. Handedness was evaluated for all participants using the Edinburgh Handedness Inventory [35].

### The cognitive reflectivity–impulsiveness questionnaire

The cognitive reflectivity–impulsiveness questionnaire [24, 36] was used to assess individual differences in reflectivity and impulsivity. This self-reported questionnaire contains 10 items and employs a four-point Likert scale with responses ranging from “I don’t agree at all” to “I agree very much” [37]. The questionnaire was developed as a substitute for the matching familiar figures (MFF) test (illustration test), which has been used to measure cognitive reflectivity and impulsivity in children [6]. The one-factor structure of the scale for this questionnaire has been supported by factor analyses [36]. Answers to all questions were compiled into a single score (with the score totaling 40, and responses from reverse items were reverted by 5—x before the summation). A high score indicated higher cognitive reflectivity, whereas a low score indicated higher cognitive impulsivity. A previous validation study using adult subjects showed that the MMF test and the cognitive reflectivity–impulsiveness questionnaire show significant correlation (r = -0.314, p < 0.01) [36].To test the validity of the cognitive reflectivity–impulsiveness questionnaire, we examined the correlation of the cognitive reflectivity–impulsiveness questionnaire scores with the impulsiveness scores of novelty-seeking from the Temperament and Character Inventory [38, 39]. The Temperament and Character Inventory scores were acquired from our sample at the same time as the cognitive reflectivity–impulsiveness questionnaire scores. We observed a significant correlation between the two parameters (r = -0.64, p < 0.01), supporting the validity of the questionnaire in parallel with the previous validation studies described above. Moreover, the internal consistency (measured using Cronbach’s coefficient α) and test-retest reliability of this questionnaire were estimated to be 0.842 and 0.827, respectively [36]. These values indicate the high reliability of the questionnaire, supporting the criterion-related validation of reflectivity and impulsivity [24].

### Assessment of psychometric measures of general intelligence

Raven’s Advanced Progressive Matrix (RAPM), one of the purest psychometric measures of general intelligence [25], was used to assess intelligence in our study in order to adjust for the well-known effect of individual psychometric measures of intelligence on brain structures [29, 40, 41]. RAPM [25] contains 36 nonverbal items requiring fluid reasoning ability. Each item consists of a 3 × 3 matrix with a missing piece to be completed by selecting the best of eight alternatives. How subjects scored on this test (number of correct answers in 30 min) was used as an index of individual psychometric measure of intelligence.

### Image acquisition and analysis

All MRI data acquisition was performed using a 3-T Philips Achieva scanner. High-resolution T1-weighted structural images (T1WIs: 240 × 240 matrix, TR = 6.5 ms, TE = 3 ms, FOV = 24 cm, slices = 162, slice thickness = 1.0 mm) were collected using a magnetization-prepared rapid gradient echo sequence.

### Preprocessing of T1-weighted structural data

Preprocessing of the structural data was performed using the Statistical Parametric Mapping software (SPM8; Wellcome Department of Cognitive Neurology, London, UK) implemented in Matlab (Mathworks Inc., Natick, MA, USA). The procedure conducted in our previous study was used [42]; using the new segmentation algorithm implemented in SPM8, T1-weighted structural images of each individual were segmented into six tissue sections. In this process, the gray matter tissue probability map (TPM) was manipulated from the original SPM8 gray matter TPM in such a way that the signal intensities of voxels (gray matter tissue probability of the default tissue gray matter TPM + white matter tissue probability of the default TPM) with intensity more than 0.25 became 0. When this manipulated gray matter TPM was used, the dura matter was less likely to be classified as gray matter (compared with when the default gray matter TPM was used), without other substantial segmentation problems. Default parameters were used in this new segmentation process with the exception that affine regularization was performed with the International Consortium for Brain Mapping template for East Asian brains. We then progressed to the diffeomorphic anatomical registration through exponentiated lie algebra (DARTEL) process implemented in SPM8. In this process, we used DARTEL-imported images of the five TPMs (extracranial space was not used because it is not consistent across subjects) from the abovementioned new segmentation method. First, we prepared a template which we had created and used in our previous studies (see [43] and [44], respectively). Using this template, we then performed DARTEL (using default parameters) for all of the subjects. The resulting images were spatially normalized to the Montreal Neurological Institute (MNI) space to yield images with 1.5 × 1.5 × 1.5 mm^3^ voxels. Subsequently, all images were smoothed by convolving them with an isotropic Gaussian kernel of 12 mm full width at half maximum (FWHM) for the reasons described below.

### Statistical analyses

We investigated rGMDs associated with individual differences in cognitive reflectivity*–*impulsivity. Statistical analyses of morphological data were then performed using VBM5 software (http://dbm.neuro.uni-jena.de/vbm/), an extension of SPM5 [45].

In the analyses, we included only voxels that showed rGMD values more than 0.05 in all subjects. The primary purpose for using GM thresholds was to cut the periphery of the GM areas so that the areas for analysis were effectively limited.

A whole-brain approach was used in this study. In the whole-brain multiple regression analysis, we tested for a relationship between cognitive reflectivity–impulsiveness (as assessed by the cognitive reflectivity–impulsiveness questionnaire) and rGMD. The age, sex, and total intracranial volume (TIV; total GM volume + total WM volume + total CSF volume) were used as additional covariates for the analysis. Furthermore, analyses were performed both with and without the RAPM score as an additional covariate in addition to the covariates used above to assess the effect of general intelligence. Of note, when total brain volume was included as a covariate in the density measures analysis, the results of the analysis showed tissue densities that could not be explained by total brain volume.

The statistical significance level in this study was set at *P <* 0.05, and corrected at the non-isotropic adjusted cluster level [46] with an underlying voxel level of *P <* 0.0025 [47, 48]. We used VBM5/SPM5 for statistical analyses (please see [49] for our rationale behind selecting the settings for the current study). The previously mentioned validation study using VBM5 [49] showed that in this non-isotropic cluster-size test of random field theory, a relatively higher cluster-determining threshold combined with high smoothing values of more than six voxels leads to appropriate conservativeness in real data. With high smoothing values, an uncorrected threshold of *P* < 0.01 seems to lead to anti-conservativeness, whereas that of *P* < 0.001 seems to lead to slight conservativeness [49]. However, there are substantial differences in the way SPM8 and SPM5 estimate the actual FWHM in the areas analyzed, and this directly affects the cluster test threshold [47, 48]. Therefore, regardless of which version is more appropriate, we believe that the conditions for this non-isotropic adjusted cluster size test shown by the previous study [49] are no longer guaranteed in SPM8. Thus, we used the VBM5/SPM5 version for statistical analyses performed in this study as in our previous studies [47, 48].

### Additional analysis of the gender effect

As described in the results section, the behavioral analysis indicated an effect of gender on cognitive reflectivity–impulsivity (Table 1). Thus, sex likely plays a role in individual differences in reflectivity, impulsivity, and brain structure. Therefore, an additional analysis of this gender effect was conducted. We investigated whether the relationship between rGMDs and the cognitive reflectivity–impulsivity scores differed between sexes (whether the interaction between sex and the cognitive reflectivity–impulsivity score affected rGMD). In the whole brain analysis, we used a voxel wise analysis of covariance (ANCOVA) in which sex difference was a group factor (using the full factorial option of SPM5). In this analysis, age, RAPM score, and total brain volume were covariates. All of these covariates, except total brain volume, were modeled so that each covariate's unique relationship with rGMD could be seen in each sex (using the interactions option in SPM8), which would allow the interaction effects of sex and the covariates to be investigated. The total brain volume was modeled so that this covariate had a common relationship with rGMD across sexes. The interaction effect between sex and the self-handicapping scale score on rGMD was assessed using t-contrasts.

**Table 1:** Statistical values of multiple regression analyses between the cognitive reflectivity–impulsiveness score and other psychological variables.

Variables	P value	T value	Regression coefficient (β)
Age	0.66	-0.44	-0.05
Sex (Female = 1, Male = 0)	0.01	-2.67	-1.08
RAPM	0.40	0.85	0.05
(Intercept)	<0.01	10.03	27.93

Note: RAPM = Raven’s Advanced Progressive Matrices

## Results

### Basic data

**Fig 1:**
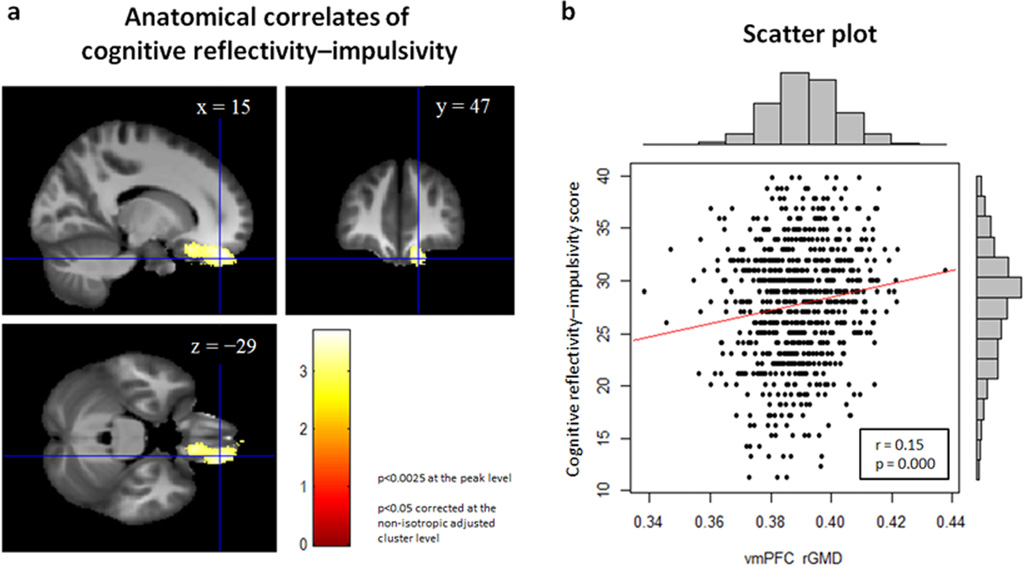
Anatomical correlates of cognitive reflectivity–impulsiveness. (a) The region of correlation is overlaid on a sagittal section (top left), a coronal section (top right), and an axial section (bottom left) of the skull stripped image of the averaged normalized T1-weighted structural images of a portion of the subjects that participated in this study. The red–yellow color scale indicates the T score of the positive correlation between rGMD and the cognitive reflectivity–impulsiveness score. rGMD was positively correlated with individual cognitive reflectivity–impulsiveness in a cluster in the medial part of the ventral prefrontal cortex (vmPFC). Results are shown with *P* < 0.05, corrected for multiple comparisons at the non-isotropic adjusted cluster-level with an underlying voxel-level of *P* < 0.0025, uncorrected. (b) A scatterplot between the cognitive reflectivity–impulsiveness score and the mean rGMD value in the significant cluster in (a) is shown for visualization purposes only. The X-axis indicates the mean rGMD value, and the Y-axis indicates the cognitive reflectivity–impulsiveness score. The upper histogram indicates the distribution of the mean rGMD value, and the right histogram indicates the distribution of the cognitive reflectivity–impulsiveness score. The distribution of these two parameters shows a significant positive correlation.

Table 2 shows the average and standard deviation (SD) of age, RAPM scores, and cognitive reflectivity–impulsiveness among subjects. A distribution of the cognitive reflectivity–impulsiveness score is indicated in Fig. 1 (right side of the figure).

**Table 2:** Demographic variables of the study participants.

Measure	Mean	SD
Age	20.70	1.84
RAPM	28.63	3.73
Cognitive reflectivity–impulsiveness	27.77	5.9

Note: RAPM = Raven’s Advanced Progressive Matrices

A multiple regression analysis with cognitive reflectivity–impulsiveness score as the dependent variable and age, sex, and RAPM score as independent variables revealed that females showed significantly lower cognitive reflectivity–impulsiveness scores (Table 1).

### Correlation between rGMD and cognitive reflectivity–impulsiveness

We investigated the association between rGMD and individual differences in cognitive reflectivity–impulsiveness. A multiple regression analysis including age, sex, RAPM score, and TIV revealed that the cognitive reflectivity–impulsiveness score was significantly and positively correlated with the rGMD in the vmPFC (peak MNI coordinates x, y, z = 15, 47, -29; peak t value = 3.70; cluster size = 1851; P < 0.001, corrected for multiple comparisons at the non-isotropic [non-stationary] adjusted cluster level with a cluster-determining uncorrected threshold of P < 0.0025; Fig. 1).

### Effects of the RAPM on the VBM results

In order to confirm the effects of the RAPM on VBM results, we conducted a VBM analysis without RAPM in the model. Positive correlations were still observed between rGMD and vmPFC with the use of the same statistical threshold as that described above (peak MNI coordinates x, y, z = 15, 47, -29; peak t value = 3.72; cluster size = 1962). In addition, we assessed brain regions, which correlated with RAPM, by focusing on the RAPM regressor. However, no brain regions were identified using the same statistical threshold as that described above. At the behavioral level, intelligence did not affect cognitive reflectivity-impulsivity (see the basic data section). In addition, intelligence did not affect the VBM analysis results. Thus, we concluded that general intelligence did not significantly impact the neural basis of cognitive reflectivity-impulsivity. This result is not consistent with those of previous studies, which identified the relationship between general intelligence and brain structure [41]. This discrepancy may be because of the sample used in this study; the participants were all high-achieving university students; thus, the general intelligence may not vary significantly thereby resulting in weak statistical results.

### Effects of the gender

The ANCOVA using data from both sexes revealed that there were no interaction effects between the score on the cognitive reflectivity–impulsivity questionnaire and gender on rGMD.

## Discussion

In this study, we demonstrated that a higher cognitive reflectivity–impulsiveness score was associated with more rGMD in the vmPFC.

### The vmPFC could act as a mediator between reflective and impulsive systems

Structural differences in the vmPFC can determine how information from the impulsive and reflective systems is utilized. Previous studies on the “somatic marker hypothesis” suggest that the vmPFC plays an important role in switching between impulsive and reflective strategies [21, 22]. In addition, the vmPFC has strong relationships with both the DLPFC, which is categorized as a reflective system, and the limbic system, which is categorized as an impulsive system. Specifically, the vmPFC interacts with either the DLPFC [50] or the limbic system [51] depending on the requirement for reflective or impulsive thoughts, respectively. Therefore, the vmPFC could act as a mediator between reflective and impulsive systems. This interpretation aids in understanding the discrepancy of the vmPFC being categorized as a reflective system in some studies but as an impulsive system in others [5, 20]. The vmPFC may be neither impulsive nor reflective; rather, it might serve as an integration area for information received from both the reflective and impulsive systems. Thus, our second hypothesis—the vmPFC forms the neural basis of cognitive reflectivity*-*impulsivity—was supported.

### Another interpretation of our results; complex information processing and the vmPFC

As an alternative interpretation of our results, an individual’s preference for reflective thoughts could result in structural differences in the vmPFC. The vmPFC is involved in complicated information processing, such as self-control and situational comprehension [50], and in high-level processing of information related to social interaction [52, 53]. It is possible that information processing becomes more complicated with increasing reflectivity, and that this would require more activation of the vmPFC. Finally, this increase in activity might also result in structural changes in the vmPFC.

### Divergence between previous results and our current findings

Lastly, there is some divergence between the results reported the literature and our current findings. Specifically, previous studies revealed a significant negative correlation between impulsivity and the lateral OFC [18, 19]. This is in contrast to findings that revealed a significant positive correlation between cognitive reflectivity and the vmPFC. This discrepancy might reflect the difference between the psychiatric concept of impulsivity and the psychological concept of cognitive reflectivity*–*impulsivity. The previous studies used an impulsiveness scale (the BIS-11 questionnaire) that was based on psychiatric measures [18]; unlike the concept of cognitive impulsiveness in psychology [12, 13], impulsiveness in psychiatry indicates inappropriate behaviors and does not consider risk [11]. Therefore, the BIS-11 questionnaire used in previous studies [54, 55] might be risk insensitive. Further, it has been suggested that the medial OFC (vmPFC) and lateral OFC have different functions [56]. In particular, the lateral OFC responds to risk [57]. Thus, the relationship that was found in the previous study between a smaller lateral OFC and impulsivity [18] is likely to be related to risk insensitivities that result in inappropriate behaviors.

### Effect of gender

At the behavioral level, we observed a gender effect in that the average cognitive reflectivity–impulsivity score was higher for females compared to males. However, we did not observe a gender effect at the brain structure level. Thus, the neural basis of cognitive reflectivity–impulsivity may be common between genders.

### Limitation

In this study, we analyzed data from 776 participants; such a large sample enabled detection of even small associations between brain structure and cognitive reflectivity-impulsivity. Associations between brain structure and personality traits have reported weak but significant relationships [42]. However, to find a more extensive relationship between brain function and cognitive reflectivity-impulsivity, investigating only brain structure would not be sufficient. Thus, more research using different approaches, such as a multivariate study or a resting connectivity study, would be beneficial.

### Future directions

One possible direction that can be pursued in future studies is the relationships among individual differences in reflectivity, impulsivity, and brain structure. This possibility focuses on other impulsiveness measurements based on the fact that other impulsiveness questionnaires exhibit different relationships with brain structures [18]. If this is the case, then our understanding of what a questionnaire actually measures might be clarified by its relationship with specific brain structures. In addition, one might think that cognitive reflectivity*–*impulsivity is not on a common axis. Thus, modeling cognitive reflectivity*–*impulsivity separately and determining its correlation in a brain structure will be a promising way to better understand cognitive reflectivity*–*impulsivity.

## Conclusion

To the best of our knowledge, this is the first study investigating associations between brain structure and cognitive reflectivity*–*impulsivity, and our results provided direct neurobiological identification of the brain structures that were associated with cognitive reflectivity*–*impulsivity. Specifically, we demonstrated a significant positive correlation between rGMD in the vmPFC and the cognitive reflectivity–impulsiveness scores. This finding suggests that the vmPFC may bridge the impulsive and reflective systems in the brain.
